# The Effect of Fibrillation, Semi-Dry Pressing, and Surface Treatment on the Barrier Properties of Water Molecules and Oxygen on Food Packaging Paper

**DOI:** 10.3390/polym16131892

**Published:** 2024-07-02

**Authors:** Yuqing Duan, Shumei Wang, Tingting Xu, Huiyang Bian, Hongqi Dai

**Affiliations:** Jiangsu Co-Innovation Center of Efficient Processing and Utilization of Forest Resources, Nanjing Forestry University, Nanjing 210037, China; dyq@njfu.edu.cn (Y.D.); wsm@njfu.edu.cn (S.W.); salyting@njfu.edu.cn (T.X.); hybian1992@njfu.edu.cn (H.B.)

**Keywords:** fibrillation, semi-dry pressing, surface coating, food packaging, barrier property

## Abstract

The characteristics of fiber morphology and paper structure are critical to the barrier properties of food packaging paper. Herein, this study aimed to use pulp fibrillation, paper semi-dry pressing and carboxymethyl starch (CMS) coating to flatten the fibers, which were formed on the paper surface with good barrier properties due to the tight bond between fibers. The results showed that the permeability of paper was reduced by 87.56%, from 81.44 μm/Pa·s to 10.13 μm/Pa·s after the pulp fibrillation treatment (60 °SR). Moreover, semi-dry pressing treatment contributed to decreasing the water vapor transmission coefficient (WVP) by 50.98% to 2.74 × 10^−10^ g/m·s·Pa, and the oxygen permeation coefficient (OP) decreased by 98.04% to 1.93 × 10^−14^ cm^3^·cm/cm^2^·s·Pa. After coating the paper surface with titanium dioxide (TiO_2_) and CMS, the WVP of the paper was further reduced to 1.55 × 10^−10^ g/m·s·Pa, and OP was reduced to 0.19 × 10^−14^ cm^3^·cm/cm^2^·s·Pa. These values were 72.27% and 99.8% lower than those of the original paper, respectively. Therefore, through pulp fibrillation, semi-dry pressing of paper, TiO_2_ filling, and surface coating with CMS, there is no need to use synthetic polymer surface film-forming agents to achieve the high barrier properties that are required for low water and oxygen molecules permeation in food packaging paper.

## 1. Introduction

Petroleum-based plastic films usually have excellent strength and barrier properties, which make them widely used materials in the field of food packaging [1,2,3]. However, due to challenges in recycling and low biodegradability, they may cause environmental threats [4,5,6]. Plant cellulose fibers are abundant in natural resources and can be used to make paper with eco-friendly, safe, and biodegradable characteristics. Therefore, they can serve as a sustainable alternative to petroleum-based plastic films [7,8,9,10]. However, cellulose-based paper has an inherent defect of a porous structure, which leads to a poor barrier for oxygen and water vapor. This structure also makes the contents easily susceptible to deterioration in food storage due to exposure to oxygen, resulting in protein deterioration or water dehydration [11,12]. Hence, functional food packaging paper has emerged as a viable alternative material to polymer plastic film, making it a highly intriguing research topic. In this regard, the significant improvement of the barrier properties to oxygen and water molecules is considered a key factor in enhancing the gas barrier properties of paper for food packaging, which has become a sustainable research focus for replacing plastic packaging [13,14,15,16].

Most of the “degradable plastic packaging materials” on the market today are made from raw materials to which starch has been added. The large pieces of plastic will break down into tiny and even invisible fragments during the environmental protection treatment process due to the fermentation of starch and bacterial decomposition. This is only a physical degradation and does not fundamentally change the issue of the challenging degradation of plastic products [17,18,19,20]. Similarly, PE film, PE laminating film, and aluminum foil on the paper surface could block gas and grease [21,22,23], but they are still difficult to degrade in an environmentally friendly way [24,25]. It is common for researchers to use bio-based polymers for surface coating. Among these, blending nanomaterials with other materials to coat the surface of paper is the most widely studied method to enhance its barrier properties. Chen Zheng [15] et al. blended nanocellulose with a chitosan solution and coated the paper surface. The coated paper had excellent water vapor and oxygen barrier properties. Khashayar Vaezi [26] et al. developed a green and biodegradable cationic starch (CS)/nano-crystalline cellulose (NCC) nanocomposite coating to enhance the water vapor and oxygen barrier properties of packaging kraft paper through surface coating. However, since paper has a porous three-dimensional network structure with large pores, the coating solution will penetrate into the pores and increase the amount of coating.

**Figure 1:**
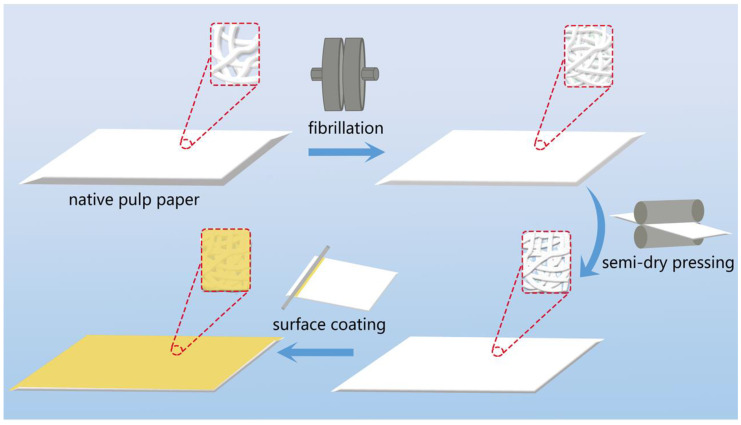
Paper barrier performance improvement process.

Based on this, by fibrillating pulp fibers, we have enhanced the plasticity of the fibers. Additionally, fibrillation could also reduce the pore structure in paper, increasing the tightness of inter-fiber bonding. To further reduce the porosity, a semi-dry pressing was used to flatten the fibers and compress the fiber voids. Finally, a mixture of carboxymethyl starch polymer (CMS) and titanium dioxide (TiO_2_) was coated onto the surface of the original paper. TiO_2_ can fill and block the voids on the paper surface [27], and CMS could act as a binder for TiO_2_ particles and form a film on the paper surface [28]. The results indicated that the excess pores of the paper were further blocked, while its barrier effect on oxygen and water vapor was greatly increased. On this basis, this paper presents a green and low-cost biomass food packaging material using paper fiber as a raw material. The main technical route is shown in Figure 1.

## 2. Materials and Methods

### 2.1. Materials

Commodity bleached coniferous pulp (Montreal, QC, Canada, Moon Brand). Titanium dioxide (TiO_2_, average particle size 200 nm) was purchased from Shanghai Macklin Biochemical Technology Co., Ltd., Shanghai, China. Carboxymethyl starch (CMS, Mw: 30,000 g/mol, DS: 0.6, food grade) was purchased from Henan Juhua Biotechnology Co., Ltd., Zhengzhou, China.

### 2.2. Methods

#### 2.2.1. Fiber Fibrillation and Based Paper Preparation

In the ZQS2-23 Valley pulper, 2% concentration of bleached coniferous wood pulp underwent viscous beating and fibrillation treatments: the degrees of fibrillation treatment for the beating degrees of 15 °SR, 30 °SR, 45 °SR, 60 °SR, and 75 °SR. The pulp was then subjected to further fibrillation treatment using an RK-2A paper machine to produce paper with a weight of 38 g/m^2^. After drying, the paper was equilibrated at 23 °C and 50% RH for 24 h, and the relevant paper property tests were conducted.

#### 2.2.2. Paper Semi-Dry Pressing

The paper was dried to 50% moisture content and semi-dry pressed by an XLS calendar with line pressures set at 0 MPa, 0.5 MPa, 1.0 MPa, 1.5 MPa, and 2.0 MPa. Subsequently, the semi-dry pressed paper was dried, equilibrated for 24 h at 23 °C and 50% RH, and then subjected to paper property testing.

#### 2.2.3. Film-Forming Agent Paper Surface Coating

With a mass ratio of surface film-forming agent (CMS, Henan Juhua Biotechnology Co., Ltd., Zhengzhou, China) to TiO_2_ (Shanghai Macklin Biochemical Technology Co., Ltd., Shanghai, China) of 5:1, the TiO_2_ dispersion and CMS were mixed uniformly using a D2004W electric stirrer to create a 5% concentration of surface coating solution. The paper was surface-coated using a CV-TB-B2 wire rod squeegee integrated coating tester at 2 g/m^2^, 4 g/m^2^, 6 g/m^2^, and 8 g/m^2^, respectively. Subsequently, it was subjected to semi-dry pressing when the paper was semi-dry, followed by drying in a DGG-9070A electrothermal constant temperature blast drying oven at 105 °C. The paper samples were treated at 23 °C and 50% RH for 24 h.

#### 2.2.4. Mechanical Properties Testing of Paper

The paper was cut into a size of 15 × 5 mm^2^, and its tensile strength was measured using the WZL-300 tensile strength machine. The YQ-Z23A paper breakage tester was used to measure the breakage resistance of paper. The paper was cut into 63 × 47 mm^2^ pieces, 4 paper samples of the same size were stacked together. The J-SLY1000A tear tester were applied to test the tearing degree of the paper. Each type of sample was repeated for 3 measurements [29].

#### 2.2.5. Paper Water Vapor and Oxygen Barrier Test

The WVP values of various papers were measured using a W3–031 Water Vapor Permeability Tester (W3/036, LABTHINK, Jinan, China) at a temperature of 37 ± 0.6 °C and a relative humidity of 50 ± 2%, with three times for each sample. The OP values of the papers were tested using a differential pressure gas permeameter (VAC-V1, LABTHINK, Jinan, China) at a temperature of 23 ± 2 °C and a relative humidity of 50 ± 10%. Three samples of each paper type were tested to calculate the average value [13].

#### 2.2.6. Paper Porosity Testing

The porosity of the paper was determined using the media saturation method [30]. Various paper samples were saturated with n-butanol. The size of the paper samples was 1 × 2 cm^2^, and they were removed after 8 h. The paper samples were sandwiched between filter paper on both sides, subjected to a specific pressure for 30 s, and then promptly weighed. Afterward, the paper samples were dried in an oven at 105 °C until completely dry. The formula for calculating the porosity of the paper is as follows:

(1) $$\varepsilon =\frac{W_{1}-W_{2}}{V\cdot \rho }$$

where ε (%) denotes the paper porosity. W_1_ (g) represents the mass of the paper sample after saturation. W_2_ (g) denotes the mass of the paper sample after drying. V (cm^3^) represents the volume of the paper sample. ρ denotes the density of n-butanol (ρ = 0.81 g/cm^3^).

#### 2.2.7. Characterization of Fiber and Paper Surface Morphology

Fiber morphology was observed using a BX41 optical microscope. The surface topography of the paper was observed using an environmental scanning electron microscope (Quanta-200, FEI, Shelbyville, KY, USA). Samples for surface topography characterization were prepared by attaching them to aluminum columns using double-sided carbon tape and then sprayed with gold. The samples were then observed and photographed for documentation. The working distance was 10–15 mm, the voltage was 10 kV, and the spot size was 3.5.

## 3. Results and Discussion

### 3.1. Degree of Fibrillation and Paper Properties

#### 3.1.1. Degree of Fibrillation and Paper Surface Morphological Structure

**Figure 2:**
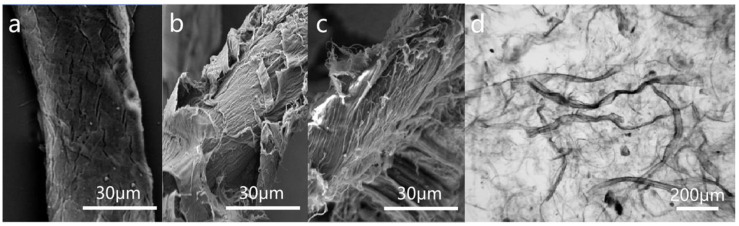
(**a**) SEM image of 15 °SR fibers (×1500); (**b**) SEM image of 45 °SR fibers (×1500); (**c**) SEM image of 60 °SR fibers (×1500); (**d**) optical microscope image of 60 °SR fibers (×100).

As shown in Figure 2, when the pulp was not beaten, the fiber diameter was about 42 μm. After mechanical treatment, the rigid structure of the pulp gradually diminished, resulting in a reduction in fiber diameter. When the degree of beating reached 60 °SR, the fiber diameter was approximately 29 μm. Due to the destruction of the rigid structure in the fiber, it absorbed water and expanded. This process broke the outer layers of the primary and secondary walls of the fiber cell wall, which weakened the interlayer binding force of the fiber microfibril, leading to the fiber end or surface filamentation.

**Figure 3:**
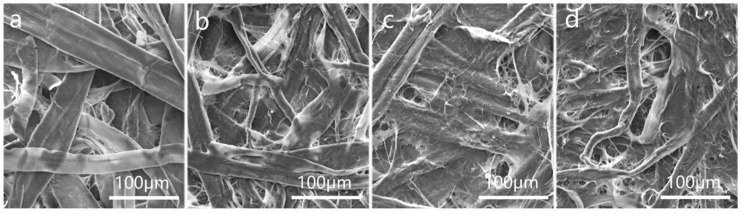
(**a**) SEM image of paper with a beating degree of 15 °SR (×400); (**b**) SEM image of paper with a beating degree of 30 °SR (×400); (**c**) SEM image of paper with a beating degree of 45 °SR (×400); (**d**) SEM image of paper with a beating degree of 60 °SR (×400).

For this reason, to guarantee the tensile and tear strength properties of packaging paper film materials, this study utilized long-fiber viscous beating and established different degrees of fibrillation (beating degrees) of the pulp at 15 °SR, 30 °SR, 45 °SR, 60 °SR, and 75 °SR, respectively. Subsequently, the pulp was made into paper with a weight of 38 g/m^2^. The strength and barrier properties of the paper were then investigated and analyzed. Figure 3a displays the surface morphology of paper from virgin pulp. The results showed a loose structure, obvious pores, and a smooth fiber surface. In Figure 3b–d, as the degree of pulp fibrillation increased, the surface of the paper became flatter due to the inter-fiber bonding. The increased degree of fibrillation led to a larger contact area between the paper fibers, thus exposing more hydroxyl groups between the fibers, which facilitates the formation of hydrogen bonds. This enhanced the cohesion between the paper fibers, making the fiber-to-fiber combination tighter. In addition, fine fibers can enhance the barrier properties of the paper by creating a “bridging” effect between the fibers. This effect reduced the average pore size of the paper and decreased the cross-sectional area of the inter-fiber voids [31,32].

#### 3.1.2. Degree of Fibrillation and Mechanical and Barrier Properties of Paper

**Figure 4:**
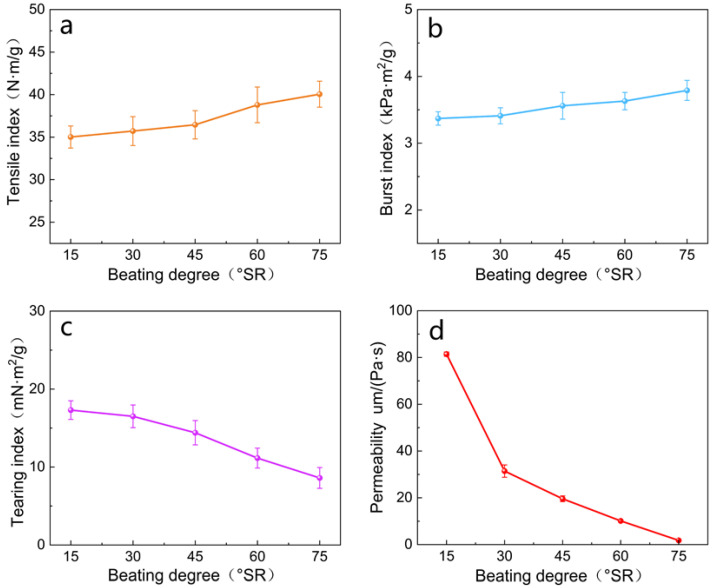
(**a**) Influence of the degree of fibrillation on the tensile index of paper; (**b**) influence of the degree of fibrillation on the burst index of paper; (**c**) influence of the degree of fibrillation on the tearing index of paper; (**d**) influence of the degree of fibrillation on the paper’s permeability.

The strength and barrier properties of 38 g/m^2^ paper with different beating degrees were analyzed. As shown in Figure 4a,b, the tensile index and burst index of paper gradually increased with the increase in the degree of pulping. The tensile index of unbeaten pulp paper was 35.02 N⋅m/g, and the burst index was 3.37 kPa⋅m^2^/g. When the degree of beating was 60 °SR, the tensile index of the paper was 38.79 N·m/g, which was 10.77% higher than that of the unbeaten pulp paper; the burst index was 3.63 kPa·m^2^/g, which was 7.72% higher. Additionally, as the specific surface area of the fibers increased, the bonding area between the fibers also increased, leading to a higher number of hydroxyl hydrogen bonding combinations between the fibers [33,34]. Consequently, the tensile strength and breaking strength of the paper increased. Figure 4c illustrates that the tear index of paper decreased as the degree of fibrillation increased. When the beating degree was 60 °SR, the tearing index of the paper was 11.15 mN·m^2^/g, which was 35.55% lower than that of the unbeaten pulp paper. This was primarily attributed to the increase in fibrillation degree and the decrease in the average length of pulp fibers. Figure 4d shows the relationship between pulp beating degree and paper permeability. As the beating degree increased, the paper permeability gradually decreased. The permeability was 10.13 μm/Pa·s when the degree of beating was 60 °SR, which was 87.56% lower than that of the unbeaten pulp paper. This was because as the degree of beating increased, the fibers became more fully moistened and swollen, enhancing their flexibility and bonding force. This increased the bonding area of the paper, made the pores between the fibers smaller, and reduced permeability.

### 3.2. Paper Semi-Dry Pressing and Paper Barrier Properties

**Figure 5:**
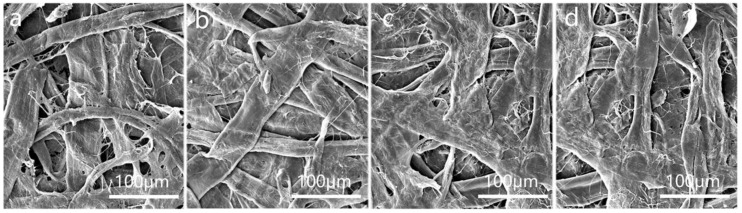
(**a**) SEM image of paper with semi-dry press of 0.5 MPa (×400); (**b**) SEM image of paper with semi-dry press of 1.0 MPa (×400); (**c**) SEM image of paper with semi-dry press of 1.5 MPa (×400); (**d**) SEM image of paper with semi-dry press of 2.0 MPa (×400).

Fibers are mechanically finely fibrillated, which not only makes the coarse fibers filamentous but also greatly reduces the inherent rigidity of the fiber structure, making the fibers soft and malleable [35]. When the paper, with a water content of 50%, was semi-dry pressed with a certain linear pressure, the radial height of the fibers and the transverse expansion, thereby reducing the cross-sectional area of the pores between the fibers. This may block the pore channels, resulting in a denser structure of the paper [36,37]. Figure 5 shows the surface morphology of the paper when line pressures were 0.5 MPa, 1.0 MPa, 1.5 MPa, and 2.0 MPa. According to the figure, the degree of fiber flattening increased significantly with line pressure, resulting in the fiber diameter increasing to about 55 μm. After the semi-dry pressing treatment, the paper surface became flatter because of a close arrangement of fibers. Moreover, the pore structure was significantly reduced.

**Figure 6:**
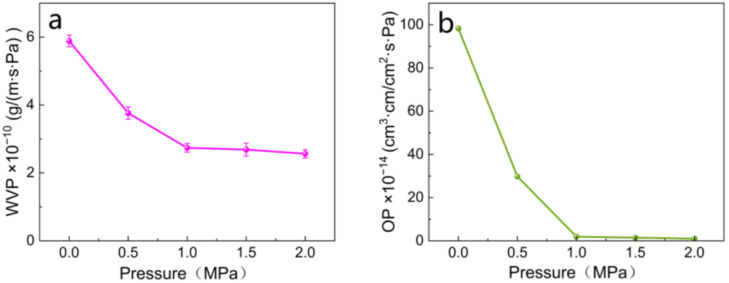
(**a**) Effect of semi−dry press on WVP of paper; (**b**) effect of semi−dry press on OP of paper.

One of the important functions of food packaging materials is to extend the shelf life of food products [38]. Enhancing the barrier properties of paper-based packaging against water vapor and oxygen is crucial because these elements can lead to alterations and degradation in food flavor. The effect of semi-dry pressing at various pressures on the water vapor and oxygen barrier properties of paper can be observed in Figure 6. The WVP and OP of the paper without semi-dry pressing were 5.59 × 10^−10^ g/m·s·Pa and 98.3 × 10^−14^ cm^3^·cm/cm^2^·s·Pa, respectively. Before the pressure was 1.0 MPa, the water vapor and oxygen transmission rates of the paper were significantly reduced, with WVP reduced by 50.98% and OP reduced by 98.04% compared to the unpressed paper. Subsequently, after the pressure was reduced to 1.0 MPa, the rate of water vapor and oxygen transmission of the paper began to slow down, and the degree of reduction became less pronounced. The WVP and OP of the paper reached their lowest values at a pressure of 2.0 MPa, measuring 2.57 × 10^−10^ g/m·s·Pa and 1.03 × 10^−14^ cm^3^·cm/cm^2^·s·Pa, respectively. The barrier properties improved by 54.1% and 98.95%, respectively, compared to the unpressed paper. This indicates that higher pressure can improve the water vapor and oxygen barrier properties of the paper by enhancing inter-fiber contact, which increases the contact area and thus reduces the pore space with the improvement of its barrier properties [39]. When the pressure was 1.0 MPa, the WVP and OP of the paper were 2.74 × 10^−10^ g/m·s·Pa and 1.93 × 10^−14^ cm^3^·cm/cm^2^·s·Pa, respectively. The water vapor and oxygen barrier properties improved by 50.98% and 98.04%, respectively. The effect on the barrier properties of the paper did not vary significantly at a pressure of 2.0 MPa. These results may be due to excessive pressure during semi-dry pressing may have caused fibers to be crushed, decreasing their mechanical properties. Additionally, the paper became wrinkled and creased when the pressure was too high, affecting both its appearance and performance, as well as its applications.

In summary, a semi-dry pressing pressure of 1.0 MPa was found to be more suitable for a paper weight of 38 g/m^2^ and a paper moisture content of 50%. Through the semi-dry pressing method, the fibers were more tightly bonded leading to reduce the porosity. This confirms that semi-dry pressing improves the paper’s water vapor and oxygen barrier performance.

### 3.3. Surface Sizing and Paper Barrier Properties

In the experiment, a natural polymer carboxymethyl surface film-forming agent (CMS) was combined with TiO_2_. The TiO_2_ and the surface film-forming agent were then mixed in a mass ratio of 1:5 to prepare a surface coating agent with a solid content of 5% (*w*:*w*). This mixture was used for coating semi-dry pressed paper. Subsequently, after drying and calendaring, the influence of the surface coating amount on the water vapor and oxygen barrier properties of the paper was evaluated.

**Figure 7:**
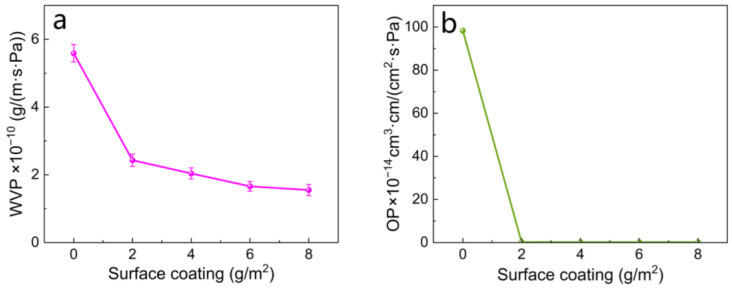
(**a**) Effect of surface coating on WVP of paper; (**b**) effect of surface coating on OP of paper.

As shown in Figure 7, the thickness of the original uncoated paper was 67 μm, and the WVP and OP were 5.59 × 10^−10^ g/m·s·Pa and 98.3 × 10^−14^ cm^3^·cm/cm^2^·s·Pa, respectively. After coating with a film-forming agent made by mixing CMS and TiO_2_, the water vapor and oxygen barrier properties of the paper improved. As the amount of coating increased, the WVP and OP of the paper decreased, indicating improved barrier performance. When the surface coating was 8 g/m^2^, the paper thickness was 71 μm, the WVP was 1.55 × 10^−10^ g/m·s·Pa, and the OP was 0.19 × 10^−14^ cm^3^·cm/cm^2^·s·Pa. These values were 72.27% and 99.8% higher than those of the original paper in terms of water vapor and oxygen barrier properties, respectively. After coating the paper surface with the film-forming agent, it was evident that the paper exhibited significantly improved water vapor and oxygen barrier properties.

**Figure 8:**
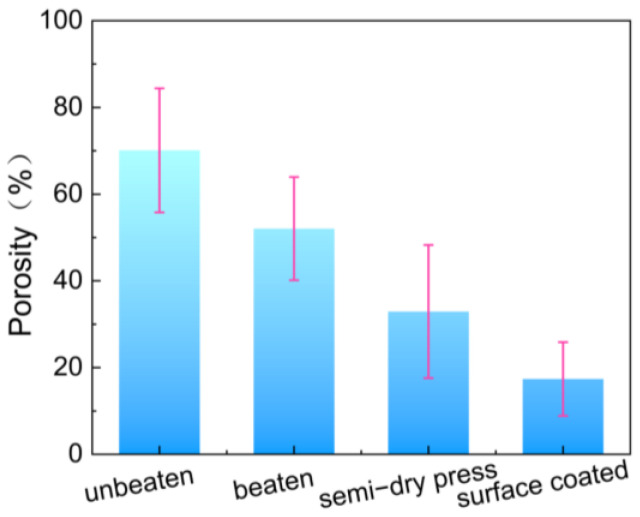
Effect of different treatment processes on paper porosity.

According to Figure 8, the porosity of unbeaten paper was 70.11%. After the fibrillation treatment, the paper porosity decreased to 52.04%. Following the semi-dry press treatment, the paper porosity further decreased to 32.92%, and after the surface coating treatment, it was reduced to 17.37%. The results indicated that the paper porosity gradually decreased with each stage of the paper processing treatment. Therefore, fibrillation, semi-dry pressing, and surface coating can effectively reduce the porosity of paper, thereby enhancing its water vapor and oxygen barrier properties.

**Figure 9:**
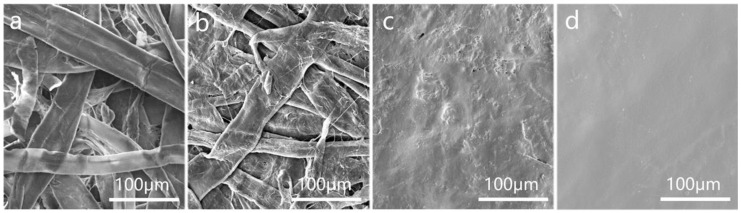
(**a**) SEM image of unbeaten paper (×400); (**b**) SEM image of semi-dry press paper (×400); (**c**) SEM image of surface coated paper (×400); (**d**) SEM image of calendared paper after coating (×400).

Figure 9 shows the surface morphology of paper with different treatments. Figure 9a shows the surface morphology of the paper without beating, while Figure 9b displays the surface morphology of the paper after semi-dry pressing. It can be observed that visible fibers and pores are present on the surface of the paper when it is not pulped treatment. However, when the paper is beaten and semi-dry pressed, the fibers become flattened, leading to an increase in the contact between fibers and subsequently reducing the pores on the paper’s surface.

Additionally, it is evident that the surface coating on pretreated paper forms a film on the paper surface (Figure 9c,d), effectively blocking the paper pores. The TiO_2_ provided covering power [27], filling the pores of the paper, and forming a layer on the paper’s surface. Sodium carboxymethyl starch has excellent film-forming properties [40] and can be coated on the paper’s surface to prepare a uniform barrier layer. This reduces the paper’s capillaries and significantly enhances its barrier performance. Unpressed paper surfaces clearly show the outline of the fibers, indicating that the paper surface is not flat. In contrast, pressed paper surfaces exhibit better film formation, resulting in a smoother, more uniform, and complete film. This enhances the paper’s water vapor and oxygen barrier performance.

## 4. Conclusions

Based on the morphology of pulp fibers and the structural characteristics of paper, this study analyzed the barrier properties of paper against water and oxygen molecules using a set of treatments, such as pulp fibrillation, semi-dry paper pressing, TiO_2_ particle filling on the paper surface, and CMS surface coating. The results are as follows: the pulp was treated with fibrillation, and the permeability of the paper decreased from 81.44 μm/Pa·s to 10.13 μm/Pa·s at a beating degree of 60 °SR, with a decrease of 87.56%. After pressing with 1.0 MPa linear pressure at 50% moisture content, the WVP of the paper decreased by 50.98%, and the OP decreased by 98.04%. The CMS dispersion containing 20% TiO_2_ particles was coated on the surface of the paper. Compared with the based paper, when the coating amount was 8 g/m^2^, the WVP and the OP of the paper decreased by 72.27% and 99.8%. Hence, the fibrillation of pulp greatly reduced the rigid structure of fiber with favorable flexibility. After semi-dry pressing, the paper made from fibrillated pulp exhibits a flattened morphology due to tighter bonding between fibers. The CMS polymer was mixed with a certain amount of TiO_2_ particles added to the surface to form a film. This film not only blocks the capillary pore structure of the paper but also completely covers the paper’s surface with a CMS polymer film. There is no need for PE coating or synthetic polymer coating to form a film. This allows the paper to satisfy the high barrier performance requirements for water and oxygen molecules. The study is expected to entirely substitute plastic food packaging with biomass-based paper food packaging to fulfill the objective of replacing plastic with paper.

## Data Availability

The data presented in this study are available on request from the corresponding author.
